# Cross Talk between Peritoneal Macrophages and B-1 Cells *In Vitro*

**DOI:** 10.1371/journal.pone.0062805

**Published:** 2013-05-08

**Authors:** Felipe Garutti Thies, Maria Fernanda Lucatelli Laurindo, Elizabeth Cristina Perez, Ronni Romulo Novaes e Brito, Mario Mariano, Ana Flavia Popi

**Affiliations:** 1 Discipline of Immunology, Department of Microbiology, Immunology and Parasitology, Universidade Federal de São Paulo, São Paulo, Brazil; 2 Universidade Paulista-UNIP, São Paulo, Brazil; Texas A&M University, United States of America

## Abstract

B-1 cells constitute a distinct B cell population with unique phenotypic and functional characteristics. They represent the main B cell population found in mouse peritoneal and pleural cavities. The communication between B-1 cells and peritoneal macrophages has been previously studied, and the effect this interaction has on macrophages has been previously described. Using an *in vitro* co-culture model, herein we demonstrated that peritoneal macrophages were able to increase survival rates and to stimulate proliferation of B-1 cells. IL-6 was also found to be important in B-1 cell survival; recombinant IL-6 increases the percentage of viable B-1 cells in culture. Furthermore, molecules involved in the IL-6 signaling pathway, such as STAT-3 and Bcl-2, were highly expressed in B-1 cells after co-culture with peritoneal macrophages. IL-6-deficient peritoneal macrophages were not able to increase B-1 cell survival, confirming the importance of this cytokine. Altogether, our results indicate a novel mechanism in which peritoneal macrophages are able to regulate the B-1 population via IL-6 secretion.

## Introduction

Homeostasis is essential for the maintenance of life. Once this equilibrium is disrupted, dynamic interactions are initiated and different components act together to orchestrate a controlled response in order to restore conditions to the previous homeostasis. The immune system is central to the maintenance of homeostasis. It is essential for minimizing damage that originates from the environment [1].

During an infection, different molecules are responsible for recognizing potential pathogens that enter the body. These receptors initiate a signaling cascade that results in the beginning of an immune response. To clear the infection completely, there must be communication between different cell types [2]. These interactions, which occur both by cell-cell contact and through secreted soluble factors, are observed in many organs and tissues. The peritoneal cavity is not an exception. Many researchers have described the peritoneal *milieu* as a dynamic environment that can respond to a variety of stimuli, ranging from BCG (Bacillus Calmette–Guérin) infection to skin transplantation, even if the stimulus is located outside of the peritoneum [3], [4]. In fact, Palos *et al* demonstrated a distinct peritoneal cell response after inoculating the footpads of mice with an irritant, showing that a distant stimulus can also affect the peritoneum cavity [5].

B-1 cells are the main B-cell population in the peritoneum of mice [6]. These cells differ from conventional B lymphocytes (B-2 cells) in many aspects, including localization, surface marker expression, antibody repertoire, developmental pathways, morphology and function [7], [8]. Moreover, Abrahao *et al* have demonstrated that the ultrastructure of peritoneal B-1 cells has no similarity to that of splenic B-2 cells [9]. B-1 cells express typical B-lineage markers, such as CD19, CD45/B220 and IgM, but unlike B-2 cells, they lack CD23 [10]. B-1 cells also express the classical myeloid marker CD11b, and the expression of CD5 characterizes two distinct B-1 subtypes: CD5^+^ cells are referred to as B-1a cells, while CD5^−^ cells are described as B-1b cells [7], [11]. Additionally, B-1 cells have the ability to secrete IL-10 without stimulation, and they use this cytokine as an autocrine growth factor [12].

Communication between B-1 cells and other immune cell subtypes has been recently elucidated. Russo *et al* described the ability of B-1 cells to modulate the cellular composition of BCG-induced pulmonary granulomatous lesions in mice [3]. Additionally, Nogueira-Martins demonstrated, in a T-cell-mediated allograft rejection model in mice, that B-1 cells permitted the infiltration of CD8^+^ T cells rather than T helper lymphocytes into the allografts [4].

B-1 cells were also described to be important for functional regulation of macrophages. Using *in vivo* models, Wong *et al.* described the influence that B-1 cells have on macrophage polarization; B-1 cells drive tumor-associated macrophages to an alternatively activated phenotype in a B16 melanoma tumor model [13]. Moreover, Popi *et al.* demonstrated that the IL-10 secreted by B-1 cells leads to a decrease in nitric oxide and hydrogen peroxide production by macrophages, which lowers their phagocytic capacity *in vitro* [14]. In agreement with these data, it was demonstrated that BALB/c mice failed to control a *Paracoccidioides brasiliensis* infection when compared to BALB/*Xid* mice, which have impaired production of B-1 lymphocytes [15]. This result was attributed to an impairment in macrophage function because of IL-10 secreted by the B-1 cells [16].

Despite the influence of B-1 cells on many immune cells, little is known about the possible role of different immune cells on the B-1 population. Based on aforementioned data, we decided to evaluate the possible influence of peritoneal macrophages on B-1 cells *in vitro.* Here, we describe that macrophages can influence B-1 cells *in vitro*, mostly by influencing their proliferation and survival.

## Methods

### Mice

BALB/c, BALB/*Xid* and C57BL/6 mice, 6–8 weeks of age, were obtained from the animal facilities of Centro de Desenvolvimento de Modelos Experimentais para Medicina e Biologia (CEDEME), UNIFESP, Brazil. C57BL/6 IL-6 knockout (KO) mice, 8 weeks old, were obtained from the University of São Paulo at Ribeirão Preto School of Medicine. In the majority of experiments the BALB/c lineage was utilized, unless indicated otherwise. This study was approved by the Research Ethical Committee (0243/07) from Universidade Federal de São Paulo, Brazil, and all efforts were made to minimize suffering.

### Peritoneal Cell Culture

B-1 cells were obtained from the peritoneal cavities of mice, as described elsewhere [17]. Briefly, peritoneal cells were collected from the abdominal cavity of mice by washing with RPMI-1640 medium (Sigma, St Louis, MO). Cells (5×10^6^ cells/ml) were dispensed onto 6-well plates (Corning Costar, Tokyo, Japan) and incubated at 37°C in 5% CO_2_ for 40 minutes. Non-adherent cells were discarded, and RPMI-1640 containing 10% fetal calf serum (Cultilab, Campinas, SP, Brazil) (R10) was added to the adherent fraction, followed by incubation at 37°C in 5% CO_2_ for 5 days with no medium renewal. After this procedure, B-1 cells comprised the main cell type in the non-adherent cell population. These non-adherent cells (B-1 cells) were collected and used in the experiments performed. The supernatants of these 5-day cultures (peritoneal cell-conditioned medium) were collected and used in some experiments. The adherent fraction of these cultures was also used as a source of macrophages (Figure S1).

### Phenotypic Analysis of Non-adherent and Adherent Fractions of Peritoneal Cell Cultures by FACS

Cells from non-adherent or adherent fractions of the culture described above were collected, and their phenotype was evaluated by fluorescence activated cell sorting (FACS). These cells were stained with the following antibodies: floating cells were stained with allophycocyanin (APC) labeled anti-mouse CD19 and fluorescein-isothiocyanate (FITC) labeled anti-mouse CD23. The adherent fractions of BALB/c and BALB/*Xid* mouse cultures were stained with FITC labeled anti-mouse CD19, phycoerythrin (PE) rat anti-mouse CD11b and anti-mouse F4/80 biotin-conjugated antibodies. After washing, these cells were also stained with streptavidin APC-conjugated antibodies. Anti-CD19, CD23, CD11b and streptavidin APC-conjugated antibodies were from BD Biosciences (Pharmingen, San Diego, CA). Anti-F4/80 antibody was from Invitrogen (Life Technologies, Carlsbad, CA). At each step, cells were maintained for 25 minutes at 4°C protected from light. Fifty thousand events were acquired on a BD® FACSCalibur using CELLQUEST software (BD Biosciences, Mountain View, CA), and data were analyzed using FlowJo® software (Tree Star).

### B-1 Cells and Macrophage Co-cultures

B-1 cells were obtained as described above and cultured in three different ways: B-1 cells alone (1×10^6^ cells - in fresh R10 media); B-1 cells (1×10^6^ cells)+peritoneal adherent cells (1×10^5^ cells - in fresh R10 media) or B-1 cells (1×10^6^ cells)+peritoneal-conditioned medium (B-1 cells+peritoneal cell-conditioned medium). Cells were obtained from wild type mice (BALB/c), BALB/*Xid* or IL-6 KO mice, as mentioned in each experiment. Peritoneal-conditioned medium was also obtained from these animals. B-1 cells were either co-cultured with wild type or IL-6 KO derived conditioned medium. Alternatively, B-1 cells were cultured with peritoneal cell-conditioned medium derived from both mouse strains or co-cultured in a transwell with the macrophages.

### Viability Analysis of B-1 Cells by FACS

B-1 cells were washed and stained as described above for quantification of the CD19^+^CD23^−^ population. After staining for cell surface markers, isotonic propidium iodide (PI) (Sigma, ST Louis, MO) solution (10x) was added to each tube (final concentration 1x) and incubated for 1 minute. Twenty thousand events were acquired on a BD® FACSCalibur using CELLQUEST software (BD Biosciences, Mountain View, CA).

### CFSE-based Proliferation Assay

B-1 cell proliferation was measured using CFSE (Carboxyfluorescein diacetate, succinimidyl ester - Molecular Probes, Eugene, OR, USA) staining. Cells were counted, and 2×10^6^ cells were resuspended in RPMI media and labeled with 5 µM CFSE at 37°C for 15 minutes. Cells were washed with RPMI 1640 supplemented with 10% FBS and resuspended in R10. Cells were cultured in 6-well round bottomed plates (1×10^6^/well for each time point) for 24 and 72 hours at 37°C and 5% CO_2_. Cells were then harvested, washed with 100 µL PBS with 1% BSA and stained with peridinin chlorophyll protein (PerCP) anti-mouse CD23 and APC anti-mouse CD19 (BD Pharmingen, San Diego, CA) for 25 minutes at 4°C. Cells were then washed with PBS-1% BSA and resuspended in PBS. Fifty thousand events were acquired and analyzed on a BD FACSCalibur using CELLQUEST software (BD Biosciences, Mountain View, CA).

### IL-6 Quantification in Culture Supernatant

Supernatants from different co-cultures performed as described above were collected and stored at −80°C. Later, they were thawed, and IL-2, IL-4, IL-6, IFN-γ, TNF-α, IL-17 and IL-10 were detected using the BD CBA Mouse Th1/Th2/Th17 Cytokine Kit (BD Biosciences, CA, USA). Briefly, 25 µL of each sample was added to 175 µL of capture beads specific for the cytokines listed above and PE labeled secondary antibodies. Samples were incubated for 2 hours at room temperature in the dark. Two-color flow cytometric analysis was performed using FACS Canto II (BD Biosciences, Mountain View, CA) and analyzed using CBA analysis software.

### Recombinant IL-6 and Anti-IL-6 Treatments

B-1 cells were obtained as previously described, counted and cultured alone, with 200, 400 or 800 pg/ml of recombinant IL-6 (BD Biosciences, CA, USA). After 24 and 72 hours, the phenotype and viability of B-1 cells were assessed as previously described. Alternatively, B-1 cells were treated for 24 hours with anti-IL-6 before or after co-culture with macrophages (BD Biosciences, CA, USA). The viability of B-1 cells was analyzed by PI staining.

### Preparation of Cellular Extracts for Western Blot

B-1 cells were cultured in conditions mentioned above for 24 hours. After this treatment, cells were lysed in hypotonic buffer (50 mM Tris-HCl pH 7.4, 100 mM NaCl, 50 mM NaF, 0.5% NP-40, 1 mM NaVO_4_, 100 mM PMSF, 10 µg/mL leupeptin and 10 µg/mL aprotinin) for 20 minutes on ice. Cell lysates were centrifuged at 14,000 rpm for 15 minutes at 4°C and the supernatants were used for Western blotting analysis. Extracts were stored at −70°C prior to use, and the protein content was determined by the Bradford method [18].

B-1 cellular extracts were fractionated by SDS–PAGE with 10% acrylamide under reducing conditions. The proteins were transferred to nitrocellulose membranes (BioRad, Hercules, CA, USA), followed by blockage of free sites with TBS containing 5% skim milk. Anti-pSTAT3 (Cell Signaling Technology, Beverly, MA, USA), STAT3 (Santa Cruz Biotechnology, Santa Cruz, CA, USA), anti-Bcl2 (Calbiochem, Merck, Darmstadt, Germany) and anti-β actin (Sigma, St Louis, MO) were used as primary antibodies, and peroxidase-conjugated goat anti-mouse IgG (Sigma, St Louis, MO) or anti-rabbit IgG (BioRad, Hercules, CA, USA) was used as a secondary antibody. The reactions were developed with a chemiluminescence ECL kit (Amersham Pharmacia, Uppsala, Sweden).

### Statistics

All data represent at least three independent experiments and are expressed as the mean ± the standard deviation. Statistical comparisons were made by the analysis of the variance and by Tukey’s or Bonferroni’s post-tests. Differences that present p-values of less than 0.05 were considered statistically significant.

## Results

### The Presence of Macrophages Increases B-1 Cell Survival and Proliferation *in vitro*

Considering that B-1 cells are able to regulate some macrophages [13], [14], we decided to evaluate if the presence of peritoneal macrophages would induce changes in the B-1 cell population *in vitro*. To evaluate this question, B-1 cells and macrophages were obtained from peritoneal cell cultures, as described above (Figure S1).

**Figure 1:**
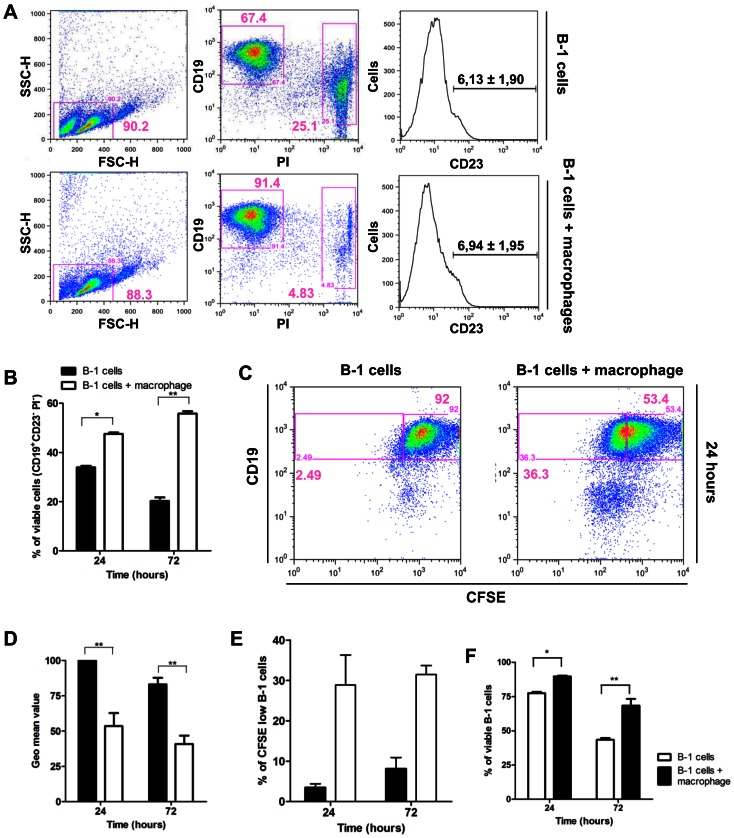
B-1 cells have their survival and proliferation increased when co-cultivated with peritoneal macrophages. (A) Schematic representation of gate strategy after 24 hours of culture. Expression of CD19 and PI were evaluated on lymphocyte and dead cell gate (left column). CD19^+^PI^−^ cells (middle column) were analyzed for CD23 expression (right column), and CD23^+^ population was excluded from all analysis. B-1 cells cultivated alone (upper panel) or with peritoneal macrophages (lower panel) are shown. (B) Graph represents percentage of viable B-1 (CD19^+^CD23^−^PI^−^) cells cultivated alone or with peritoneal macrophages in 24 and 72 hours. (C) Representative dot plots of B-1 cells stained with CFSE cultured alone (B-1 cells) or with peritoneal macrophages (B-1 cells+macrophage), after 24 hours. (D) Graph represents geometric mean values of CFSE staining after 24 and 72 hours for B-1 cultivated alone or with peritoneal macrophages. (E) Graph represents percentage of CFSE low B-1 cells when cultured alone or with peritoneal macrophages.

B-1 cells were cultured alone or in the presence of peritoneal macrophages for 24 or 72 hours, and the viability and proliferation rate of B-1 cells were analyzed. Figure 1A shows a schematic representation of the gate strategy used throughout the figures. Using forward (FSC) and side scatter (SSC) dot plots, 30,000 events were acquired from gated lymphoid and dead cells (Figure 1A, left panel). From this population, B-1 cells (CD19^+^PI^−^) made up 67.4% and 91.4% of the viable cells when cultured alone or in the presence of peritoneal macrophages, respectively (Figure 1A, middle panel). To exclude any possibility of conventional B cell contamination, CD23 expression by CD19^+^PI^−^ cells was analyzed (Figure 1A, right panel) and CD23^+^ cells were excluded from all analyses. Figure 1B shows that a significantly higher percentage of B-1 cells (CD19^+^CD23^−^PI^−^) were alive when cultured in the presence of peritoneal macrophages for 24 or 72 hours than those B-1 cells cultured alone. Similar results were obtained when B-1 cells were purified by cell sorting (99% of purity –Figure S3) and cultured together with peritoneal macrophages for 24 hours (Figure 1F).

As Almeida *et al* demonstrated, B-1 cells are able to proliferate *in vitro* [17], and we wondered if contact with peritoneal macrophages could modify the B-1 cell proliferation index. Thus, B-1 cells were stained for CFSE and cultured alone or in the presence of peritoneal macrophages. Figure 1C shows CFSE staining of B-1 cells cultured alone or with peritoneal macrophages for 24 or 72 hours. Analysis of CFSE-labeled cells showed that peritoneal macrophages significantly increased B-1 cell proliferation *in vitro* (Figure 1D and E). On Figure 1D, the geo mean value of B-1 cells cultured alone after 24 hours were used as reference for the analysis B-1 cells cultured alone for 72 hours and with peritoneal macrophage for 24 and 72 hours. Figure 1E shows the percentage of B-1 cells with low CFSE staining.

Taken together, these data indicate that macrophages influence the survival and proliferation of B-1 cells *in vitro*.

### Soluble Factors Produced by Peritoneal Macrophages are Sufficient to Modify B-1 Cell Survival *in vitro*

**Figure 2:**
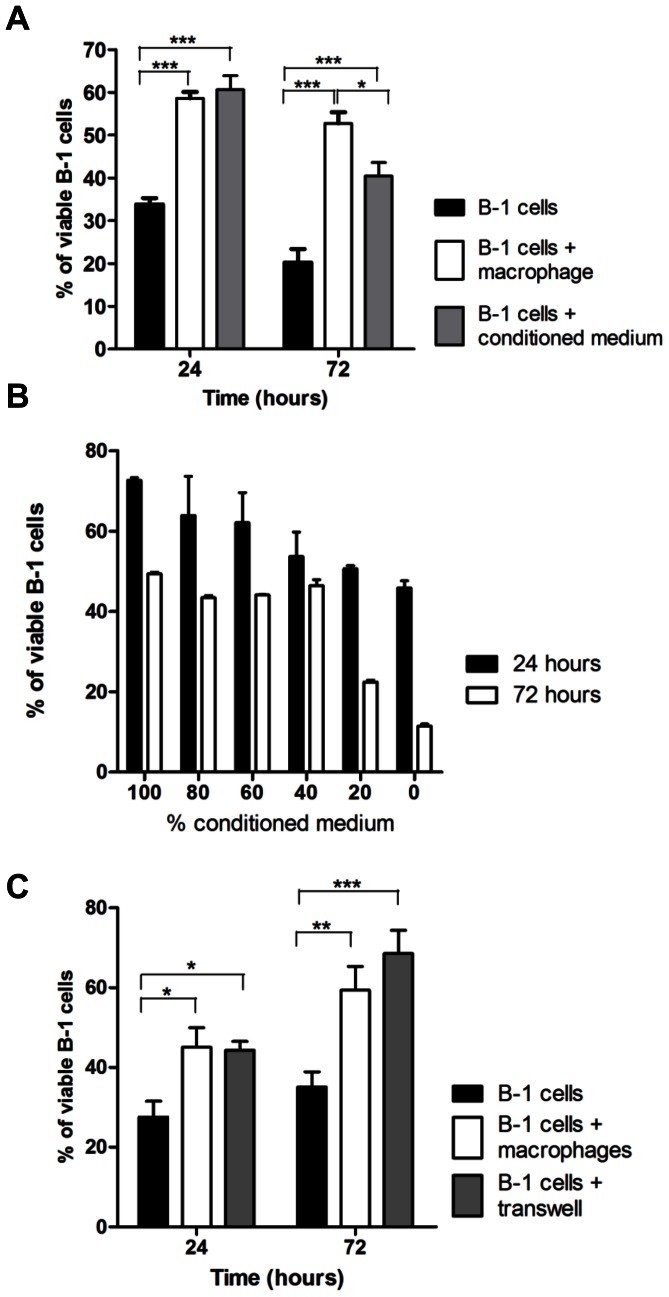
A soluble factor is essential for B-1 cell survival *in vitro*. (A) Graph represents percentage of viable B-1 cells when cultivated alone, together with peritoneal macrophages or with conditioned medium, after 24 and 72 hours. (B) Viability was measured when B-1 cells were cultivated with different concentration of the conditioned medium. (C) Survival of B-1 cells was evaluated when they were cultured on transwells placed on peritoneal macrophages. *p<0.05, **p<0.01, ***p<0.001.

Considering that B-1 cell proliferation and survival are increased in the presence of peritoneal macrophages, we hypothesized that soluble factors secreted by the peritoneal macrophages could have an important role in the observed phenotype. To investigate this hypothesis, B-1 cells were cultured as indicated: in fresh medium (B-1 cells), in the presence of peritoneal macrophages (B-1 cells+macrophages) or with peritoneal cell-conditioned medium (B-1 cells+conditioned medium) over the course of 24 or 72 hours. As shown in Figure 2A, after both periods, we observed an increased amount of viable B-1 cells when these cells were cultured in conditioned medium compared to B-1 cells cultured alone. When compared to B-1 cells cultured with peritoneal macrophages, B-1 cells+conditioned medium had similar viability after 24 hours, and a reduction was noted after 72 hours (Figure 2A). In addition, using different percentages of conditioned medium resulted in a progressive dilution of soluble factors and a decrease in the number of viable B-1 cells in culture, in a dose-dependent manner, either after 24 or 72 hours (Figure 2B).

To confirm that a soluble factor is essential for the augmented B-1 cell survival *in vitro*, B-1 cells were cultured in the top of transwell chambers placed over peritoneal macrophages. Figure 2C shows that even without direct contact between macrophages and B-1 cells, the presence of peritoneal macrophages increased lymphocyte viability after 24 and 72 hours, as compared to B-1 cells cultured alone. Altogether, these results confirm the presence of a peritoneal macrophage-derived soluble factor that increased the survival of B-1 cells.

**Figure 3:**
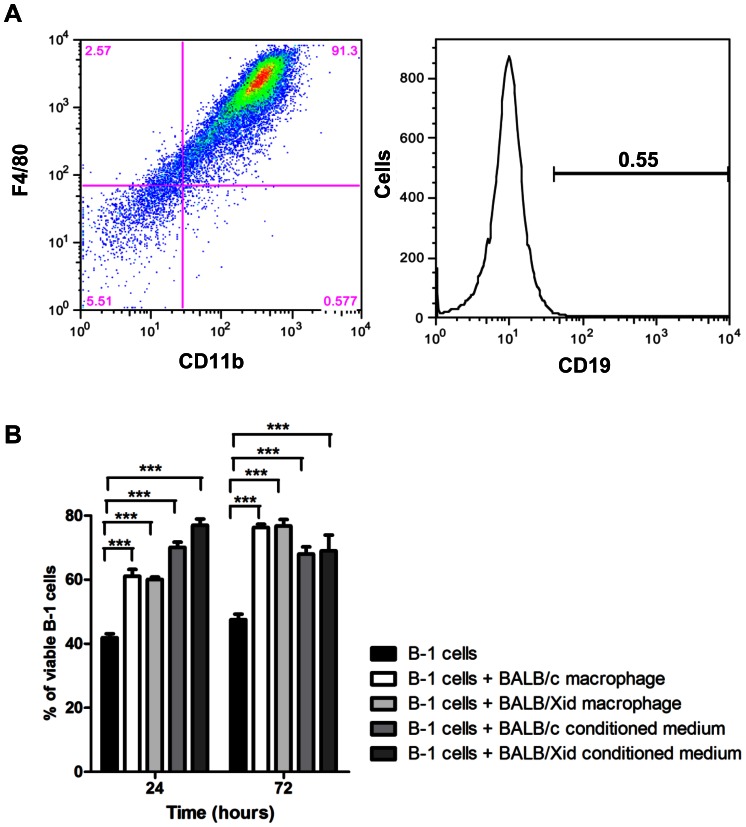
BALB/*Xid* peritoneal macrophages and conditioned medium augment B-1 cell survival. (A) Representative dot plot of BALB/*Xid* peritoneal macrophages stained with F4/80, CD11b (left panel) and CD19 (right panel). (B) Graph represents percentage of viable B-1 cells present in culture after 24 and 72 hours when B-1 cells were cultivated alone, on BALB/c or BALB/*Xid* peritoneal macrophages or with BALB/c or BALB/*Xid* conditioned medium. ***p<0.001.

To eliminate the possibility that B-1 cells present in the adherent fraction (less than 0.5% as shown in Figure S1) could maintain B-1 cell survival by producing a soluble factor, the adherent fraction of BALB/*Xid* peritoneal cell cultures was used as the source for peritoneal macrophages. Flow cytometric analysis of the peritoneal cell culture from BALB/*Xid* confirmed the deficiency of B-1 cells (Figure S2). Analysis of the BALB/*Xid* adherent fraction showed that it was mainly composed of peritoneal macrophages, similar to that previously observed in BALB/c cultures, with 91.3% of cells with the CD11b^+^F4/80^+^ phenotype (Figure 3A).

Corroborating the previous data, BALB/*Xid* peritoneal macrophages were also able to significantly increase B-1 cell survival *in vitro* after 24 or 72 hours (Figure 3B), when compared to B-1 cells cultured alone. Moreover, when B-1 cells were cultured in the conditioned medium obtained from the BALB/*Xid* peritoneal cell culture, we observed similar percentages of viable cells compared to the results with conditioned medium from BALB/c cells; both were significantly increased compared to B-1 cells cultured alone (Figure 3B).

Taken together, our results exclude the possibility of B-1 cell contamination in the adherent fraction and clearly show that soluble factors produced by peritoneal macrophages can promote B-1 cell survival *in vitro*.

### IL-6 Increases B-1 Cell Viability

**Figure 4:**
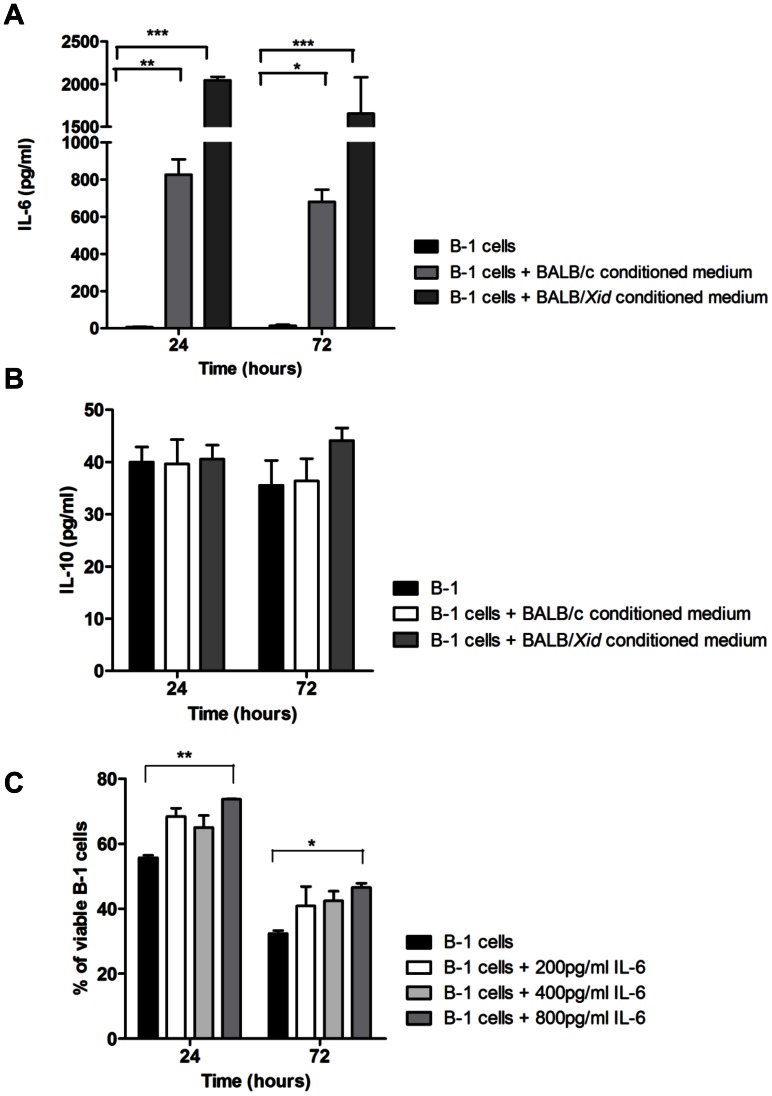
IL-6 increases B-1 cell viability. (A) IL-6 and IL-10 (B) were measured when B-1 cells were cultivated alone or with BALB/c or BALB/*Xid* conditioned medium, after 24 and 72 hours. (B) B-1 cells were cultivated alone or with 200, 400 or 800 pg/ml of recombinant IL-6, and their survival was measured. *p<0.05, **p<0.01, ***p<0.001.

Considering that macrophages produce high amounts of interleukin-6 (IL-6) and that this cytokine is responsible for lymphocyte proliferation and survival [19], we evaluated IL-6 levels in our model. Figure 4A shows higher amounts of IL-6 present when B-1 cells were cultured with conditioned medium derived from BALB/c or BALB/*Xid* than when B-1 cells were cultured in fresh medium. Other cytokines were measured (IL-2, IL-4, IFN-γ, TNF-α, e IL-17), however, none were present in significant concentrations (data not shown). IL-10 was also detected within the culture, with no difference between groups (Figure 4B). Furthermore, the role of IL-6 in sustaining the viability of B-1 cells in culture was also tested by the addition of recombinant IL-6. Despite a slight increase in the number of viable cells after the addition of 200 or 400 pg/ml of recombinant IL-6 over those cells receiving no cytokine, no significant differences were observed between these groups (Figure 4C). However, treatment of B-1 cells with 800 pg/ml of recombinant IL-6 augmented cell viability when compared to B-1 cells cultured in fresh medium (Figure 4C). Altogether, these data indicate an important role for IL-6 on B-1 cell survival.

### IL-6 Increases pSTAT-3 and Bcl-2 Expression

**Figure 5:**
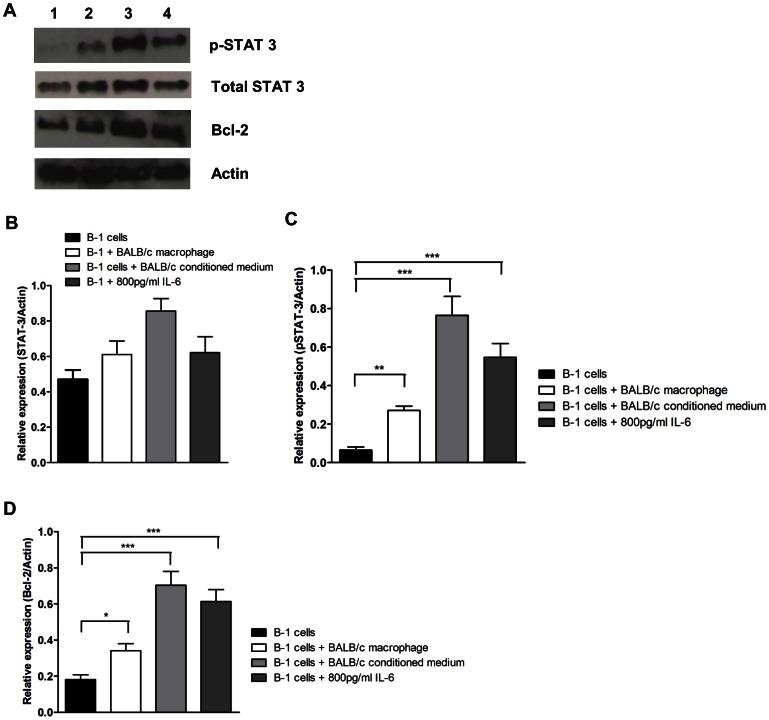
IL-6 cytokine increases pSTAT-3 and Bcl-2 expression. (A) Representative western blot of STAT-3, pSTAT-3 and Bcl-2 of B-1 cells cultivated alone (line 1), with BALB/c peritoneal macrophages (line 2), with BALB/c conditioned medium (line 3) or with 800 pg/ml of recombinant IL-6 (line 4), after 24 hours. Results are representative of 3 independent experiments. Relative expression of total (B) STAT-3, (C) pSTAT-3 and (D) Bcl-2 compared to β-actin are shown in the graphs. *p<0.05, **p<0.01, ***p<0.001.

STAT-3 phosphorylation is one of the main intracellular consequences of IL-6 binding to its receptor [19]. Therefore, we evaluated the activation of this signaling pathway in B-1 cells that were cultured in different conditions. Figure 5A shows a representative Western blot of B-1 cells cultured alone (Line 1), in the presence of BALB/c peritoneal macrophages (Line 2), in peritoneal cell-conditioned medium (Line 3) and with 800 pg/ml of recombinant IL-6 (Line 4) after 24 hours of culture. Figures 5B and C show the relative expression of STAT-3 and phosphorylated STAT-3 (pSTAT-3), compared to β-actin, in B-1 cells cultured with the previously described groups. STAT-3 expression was not significantly different for any of the groups (Figure 5B), while pSTAT-3 was increased in all groups compared to B-1 cells cultured alone. Moreover, Figure 5D shows an increase in Bcl-2, an anti-apoptotic protein that is regulated by the STAT3 transcription factor, in all treated groups when compared to B-1 cells cultured alone. Together, these data strongly support the idea that IL-6 is responsible for B-1 survival in our model, primarily through increased Bcl-2 and pSTAT-3 expression. These data reinforce the hypothesis that IL-6 participates in the survival of B-1 cells *in vitro*.

### Peritoneal Macrophages Derived from IL-6 KO Mice are Unable to Maintain B-1 Cell Viability

**Figure 6:**
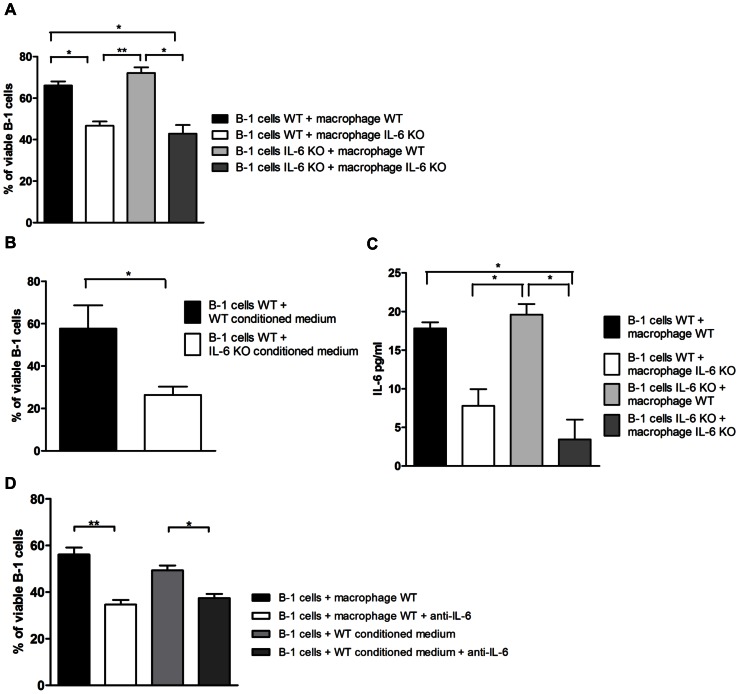
Peritoneal macrophages derived from IL-6 KO mouse are unable to maintain B-1 cells viability. B-1 cells derived from C57BL/6 wild type or IL-6 KO mouse were cultivated with wild type or IL-6 KO peritoneal macrophages (A), or with conditioned medium derived from IL-6 KO or WT mouse (B), and viability was measured after 24 hours. (C) IL-6 was measured when B-1 cells derived from wild type or IL-6 KO mouse were cultivated with wild type or IL-6 KO peritoneal macrophages. (D) Anti IL-6 was added to the culture, and B-1 cells viability was measured. *p<0.05, **p<0.01, ***p<0.001.

To confirm the involvement of IL-6 in maintaining B-1 cell survival *in vitro* and to determine the source of IL-6 in our *in vitro* model, we cultured B-1 cells derived from IL-6 KO or wild type (WT) mice together with peritoneal macrophages also derived from these mouse strains. Since the background of IL-6 knockout mouse is C57BL/6, the wild type animals used in this experiment were C57BL/6. After 24 hours, the viability of B-1 cells from IL-6 KO or WT mice was significantly reduced after being cultured with IL-6 KO peritoneal macrophages compared to B-1 cells cultured with WT macrophages (Figure 6 A). Similarly, B-1 cells cultured with conditioned medium from WT macrophages had increased viability when compared to B-1 cells cultured with IL-6 KO-derived conditioned medium (Figure 6B). Moreover, the overall IL-6 concentration was reduced when B-1 cells were cultured with IL-6 KO peritoneal macrophages (Figure 6C). These results demonstrate that macrophages are the main source of IL-6 in this system.

Finally, anti-IL-6 was added to cultures and the viability of B-1 cells *in vitro* was evaluated. When anti-IL-6 was added to the system, the viability of B-1 cells that were cultured with macrophages or with conditioned medium was statistically reduced (Figure 6D). Together, these results corroborate the importance of IL-6 production by peritoneal macrophages for B-1 cell survival.

## Discussion

The role of B-1 cells in the regulation of other immune cells has been widely reported. It has been demonstrated that B-1 cells down-regulate macrophage functions *in vitro* [14], induce macrophage polarization to an alternative phenotype [13], modulate T cell infiltration into allografts [4] and into the pancreas in mice that spontaneously develop diabetes [20], induce the differentiation of CD4^+^ T cells to become pro-inflammatory Th17 cells [21] and also have a tolerogenic function in a model of allergic reaction [22]. Not only do B-1 cells participate in physiological processes, but they also have a role in some malignancies. Perez *et al* have observed that B-1 lymphocytes can alter the properties of tumor cells required for invasiveness during metastasis [23]. Furthermore, Mussalem *et al* [24] demonstrated that B-1 cells are activated by the administration of *Propioniumbacterium acnes*, inducing the expression of molecules involved in capturing and processing antigens. These interactions with different cell types indicate that B-1 cells may have an important role in regulating different classes of immune responses, ranging from innate to adaptive roles.

Significantly less evidence is available regarding the effects of different cells on B-1 lymphocytes. We have demonstrated that melanoma cells are able to modify B-1 cells after their interaction, leading to both increased cell viability and rate of proliferation [25]. Moreover, contact with melanoma cells also increases the survival of B-1 cells after irradiation [25]. In this work, we present data showing that interaction with peritoneal macrophages also culminates in modifications of B-1 cells.

Here, we demonstrated that peritoneal macrophages were able to increase B-1 cell viability and proliferation *in vitro.* This information introduces an important new mechanism of B-1 cell survival. Early reports indicate that B-1 cells are self-renewing cells, maintaining viability through IL-10 production [12]. Production of this cytokine may be an important mechanism primarily for B-1 cell development because B-1 cells appear earlier than follicular B cells or macrophages in ontogeny [26]. Thus, the fact that B-1 cells, during early phases of development, do not depend on other cells to maintain themselves may be seen as an evolutionary advantage. Despite that, it seems reasonable that, in later phases of development, other mechanisms may act together to maintain B-1 cell viability and regulation. Indeed, immune cells are finely regulated, and normally, several mechanisms are responsible for increasing or reducing certain cell types, depending on what the organism needs to maintain homeostasis.

Moreover, we showed that regulation of B-1 cell viability occurred mainly through the production of a soluble factor by peritoneal macrophages. Because B-1 cells are able to migrate from the peritoneal cavity to an inflammatory focus [17], [27], [28], it is important that this regulation might occur at a distance. Notably, B-1 cells cultured in transwells or with peritoneal-conditioned medium, despite the increase in viability, had no increase in their rate of proliferation (data not shown). These results show that B-1 cell-macrophage contact is also important for B-1 regulation.

Considering that B-1 cells are capable of self-maintenance through production of IL-10 [12], it might be postulated that the peritoneal adherent cells were contaminated by B-1 cells. Despite the demonstration that the main population on adherent fraction are macrophages, we also excluded this possibility by culturing B-1 cells in the presence of peritoneal macrophages derived from BALB/*Xid,* mice, which have impaired production of B-1 lymphocytes [15], or in conditioned medium derived from peritoneal cells. Similar results were found between these groups. These data confirm the participation of peritoneal macrophages, and a macrophage-derived soluble factor, on B-1 cell regulation.

IL-6 is a cytokine produced by several cell types, including antigen presenting cells, such as macrophages and dendritic cells [29], and it induces B-cell proliferation [30]. Moreover, B-1 cells constitutively express high levels of IL-6 receptor [31]. All of these data strongly indicate that IL-6 could participate in B-1 cell regulation. Indeed, several experiments confirmed IL-6 participation in B-1 cell regulation. Not only was the concentration of IL-6 high in conditioned medium, but the addition of recombinant IL-6 was also able to increase B-1 cell viability. Moreover, phosphorylated STAT-3 and Bcl-2 expression was increased when B-1 cells were cultured with peritoneal macrophages or with conditioned medium. The IL-6 intracellular cascade includes phosphorylation of STAT-3 [19], which regulates other anti-apoptotic genes, such as Bcl-2 [32]. Finally, the addition of anti-IL-6 reduced B-1 cell viability. Recognizing IL-6 as a B-1 cell regulating factor may have important consequences. It is known that IL-6 plays a critical role in the pathogenesis of autoimmune diseases [33], and overproduction of IL-6 has previously been described in rheumatoid arthritis in humans [34], [35]. Additionally, the involvement of IL-6 in autoantibody production has been further supported by studies in human patients and in animal models [33]. Considering that high levels of B-1 cells have been reported in patients with Systemic Lupus Erythematosus, Sjogren’s syndrome and rheumatoid arthritis [36] and that numerous associations between expansion of this cell compartment and systemic autoimmunity have been found in murine models [37], we propose here a link between these two phenomena. In conditions in which IL-6 production is constitutively high, such as in aging [38] or in chronic inflammatory situations [39], viable B-1 cells would be augmented as well. This microenvironment would then be favorable to the development of autoimmune diseases. It has already been shown that autoimmunity is more prevalent in these situations [40]. Whether the B-1 cells of these populations are somehow altered or present in higher numbers is a question to be answered. Additionally, in NZB/W F1 mice, a mouse strain that develops an autoimmune condition accepted as a murine model of Systemic Lupus Erythematosus [41] and that has a large B-1 cell population in the peritoneal cavity [7], spontaneous production of IgG anti-DNA antibodies by splenic B cells was enhanced by IL-6 [42]. IL-6 could have the same effect on B-1 cells because they also produce anti-DNA antibody [43].

In conclusion, we have shown that peritoneal macrophages are important for the viability of B-1 cells, mainly by the production of IL-6. Extrapolating this result to in vivo, we could suggest that an increase in IL-6 levels could result in the expansion of the B-1 cells observed in some autoimmune diseases. These findings are important for the understanding of the biological function of B-1 cells and reveal an as yet unknown mechanism of B-1 cell regulation.

## Supporting Information

Figure S1 **B-1 cells and peritoneal macrophages are the main population of cultures.** (A) Analysis of non-adherent fraction of the peritoneal cells culture demonstrated that B-1 cells, characterized as CD19^+^CD23^−^ cells, comprised approximately 80% of non-adherent cells. (B) On adherent fraction, 92.2% of total cells were CD11b^+^F4/80^+^ (left panel), and less than 0.5% were CD19^+^ (right panel). These cells were used here as source of peritoneal macrophages.
(TIFF)

Figure S2 **Flow cytometry analysis of peritoneal cells culture from BALB/** ***Xid*** **.** Representative dot plot showing percentage (3.02%) of B-1 cells (CD19^+^CD23^−^) of BALB/*Xid* peritoneal cell culture.
(TIFF)

Figure S3 **Flow cytometry analysis of peritoneal cells culture after cell sorting.** Representative dot plot showing that 99% of the cells present in culture after cell sorting were B-1 cells (CD19^+^CD23^−^).
(TIFF)
