# Supplementary figures and images for: Cross Talk between Peritoneal Macrophages and B-1 Cells In Vitro
Source: PLoS One. 2013 May 8;8(5):e62805. doi: 10.1371/journal.pone.0062805 (PMC3648527; doi:10.1371/journal.pone.0062805)

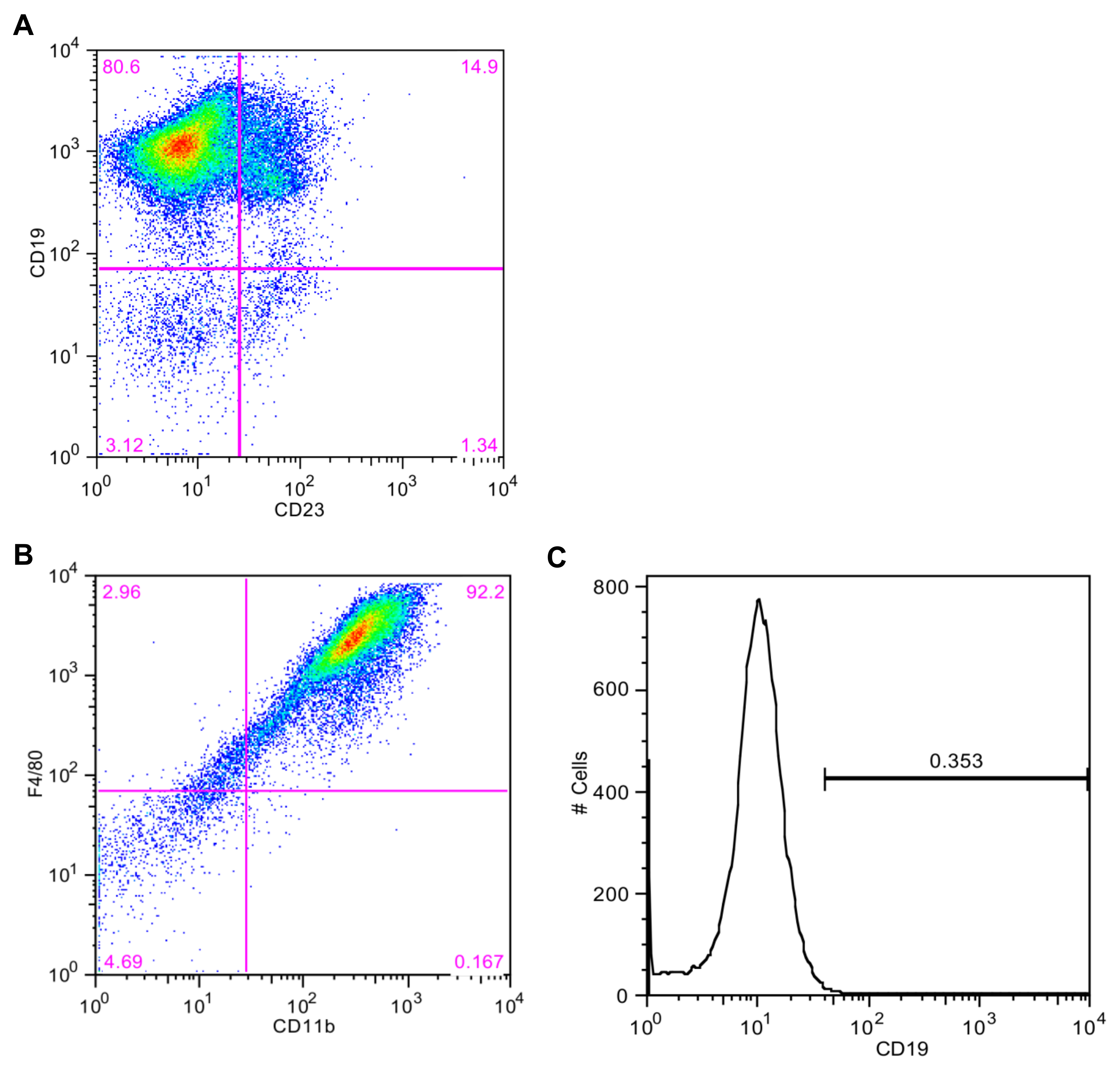

Supplement: Figure S1 — B-1 cells and peritoneal macrophages are the main population of cultures. (A) Analysis of non-adherent fraction of the peritoneal cells culture demonstrated that B-1 cells, characterized as CD19+CD23− cells, comprised approximately 80% of non-adherent cells. (B) On adherent fraction, 92.2% of total cells were CD11b+F4/80+ (left panel), and less than 0.5% were CD19+ (right panel). These cells were used here as source of peritoneal macrophages. (TIFF) [file pone.0062805.s001.tiff]

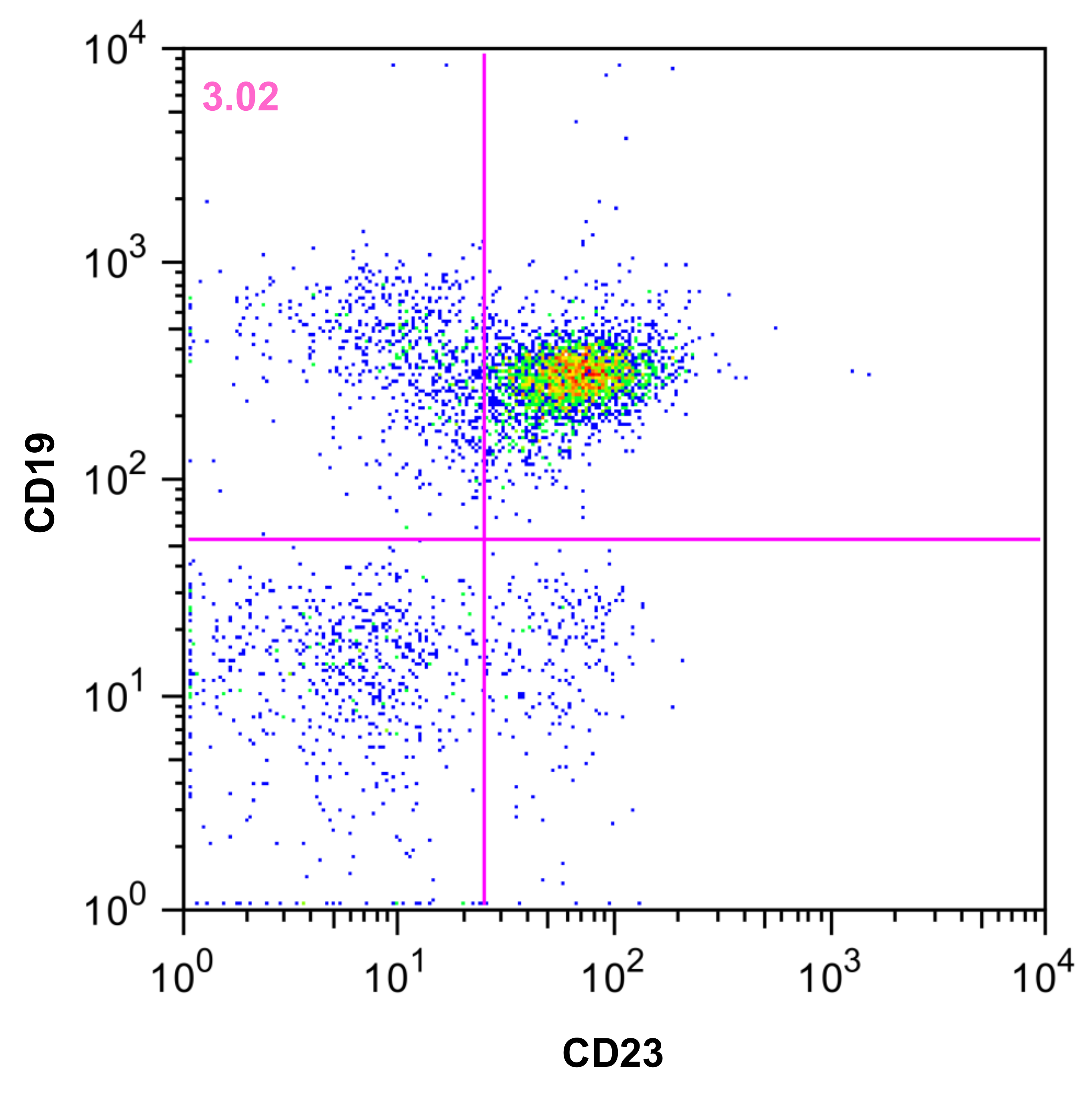

Supplement: Figure S2 — Flow cytometry analysis of peritoneal cells culture from BALB/ Xid . Representative dot plot showing percentage (3.02%) of B-1 cells (CD19+CD23−) of BALB/Xid peritoneal cell culture. (TIFF) [file pone.0062805.s002.tiff]

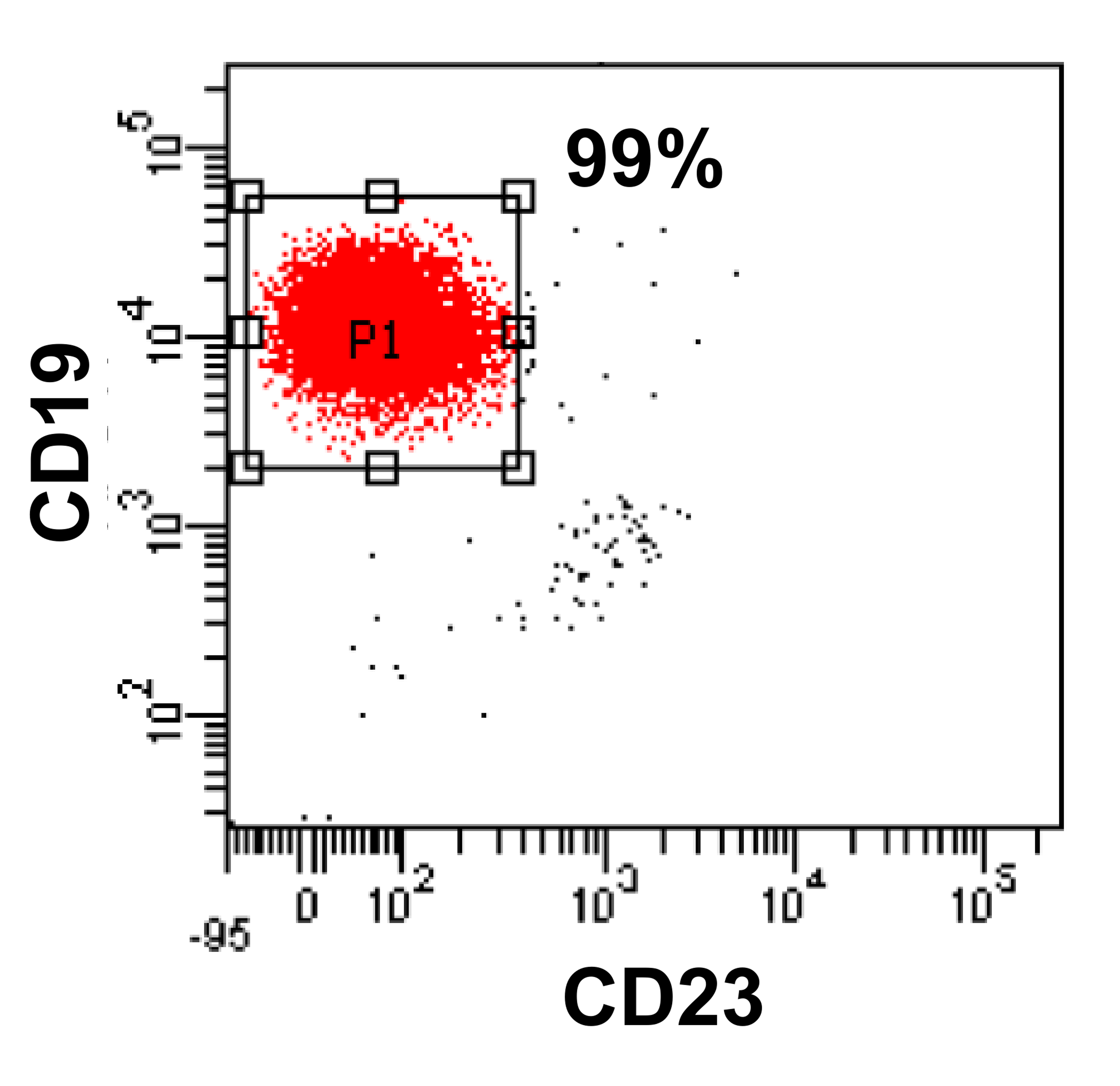

Supplement: Figure S3 — Flow cytometry analysis of peritoneal cells culture after cell sorting. Representative dot plot showing that 99% of the cells present in culture after cell sorting were B-1 cells (CD19+CD23−). (TIFF) [file pone.0062805.s003.tiff]
